# Expression and characterization of the UL31 protein from duck enteritis virus

**DOI:** 10.1186/1743-422X-6-19

**Published:** 2009-02-10

**Authors:** Wei Xie, Anchun Cheng, Mingshu Wang, Hua Chang, Dekang Zhu, Qihui Luo, Renyong Jia, Xiaoyue Chen

**Affiliations:** 1 Avian Diseases Research Center, College of Veterinary Medicine of Sichuan, Agricultural University, Ya'an, Sichuan, 625014, PR China; 2 Key Laboratory of Animal, Diseases and Human Health of Sichuan Province, Ya'an, Sichuan, 625014, PR China

## Abstract

**Background:**

Previous studies indicate that the UL31 protein and its homology play similar roles in nuclear egress of all *herpesviruses*. However, there is no report on the UL31 gene product of DEV. In this study, we expressed and presented the basic properties of the DEV UL31 product.

**Results:**

The entire ORF of the UL31 was cloned into pET 32a (+) prokaryotic expression vector. *Escherichia coli *BL21(DE3) competent cells were transformed with the construct followed by the induction of protein expression by the addition of IPTG. Band corresponding to the predicted sizes (55 kDa) was produced on the SDS-PAGE. Over expressed 6×His-UL31 fusion protein was purified by nickel affinity chromatography. The DEV UL31 gene product has been identified by using a rabbit polyclonal antiserum raised against the purified protein. A protein of approximate 35 kDa that reacted with the antiserum was detected in immunoblots of DEV-infected cellular lysates, suggesting that the 35 kDa protein was the primary translation product of the UL31 gene. RT-PCR analyses revealed that the UL31 gene was transcribed most abundantly during the late phase of replication. Subsequently, Immunofluorescence analysis revealed that the protein was widespread speckled structures in the nuclei of infected cells. Western blotting of purified virion preparations showed that UL31 was a component of intracellular virions but was absent from mature extracellular virions. Finally, an Immunofluorescence assay was established to study the distribution of the UL31 antigen in tissues of artificially DEV infected ducks. The results showed that the UL31 antigen was primarily located in the cells of digestive organs and immunological organs.

**Conclusion:**

In this work, we present the basic properties of the DEV UL31 product. The results indicate that DEV UL31 shares many similarities with its HSV or PRV homolog UL31 and suggest that functional cross-complementation is possible between members of the *Alpha*herpesvirus subfamily. Furthermore, in vivo experiments with ducks infected with UL31-defective isolates of DEV will also be of importance in order to assess the possible role of the UL31 protein in viral pathogenesis. These properties of the UL31 protein provide a prerequisite for further functional analysis of this gene.

## Background

Duck virus enteritis (DVE) is an acute and contagious disease of birds from the order *Anseriformes *(ducks, geese, and swans) [1-3]. The causative agent of the DVE is Duck enteritis virus (DEV), a member of the subfamily *Alphaherpesvirinae *[4]. As with many other herpesviruses, DVE can establish inapparent infections in birds that survive exposure to it, a state referred to as latency [5]. This makes the disease difficult to monitor and control. The genome of DEV is composed of a linear, double stranded DNA and the G+C content is 64.3%, higher than any other reported avian herpesvirus in the subfamily *Alphaherpesvirinae *[6]. There has been little information about the molecular characteristics of DEV since the disease was report in 1926. Although the molecular structure of the genome has not been reported, the DEV genomic library was successfully constructed in our laboratory [7].

During lytic infection, many herpesvirus proteins are involved in the early steps of viral maturely at the nuclear envelope, which include the UL31 of Herps simplex virus (HSV) and Pseudorabies virus (PRV) [8-11]. The UL31 protein of HSV-1 is a nuclear matrix-associated phosphoprotein stabilized by its interaction with the UL34 protein [12,13]. The two proteins interact to form a complex colocalized at the nuclear rim of infected cells, and become incorporated into virions during envelopment at the inner nuclear membrane [13-15]. With many similarities and a few differences, accumulating evidence indicates that the UL31 protein and its homology play similar roles in nuclear egress of *Alpha-*, *Beta-*, and *Grammherpesviruses *[8,14,16-20]. However, there is no report on the identification and characterization of the UL31 gene product of DEV.

In the present study, the UL31 gene was amplified from the genome of DEV and successfully expressed in a prokaryotic expression system. We prepared polyclonal antiserum which allowed identifying and characterizing the UL31 gene product of DEV. We found that the UL31 gene was transcribed most abundantly during the late phase of replication, and the UL31 protein was approximately 35 kDa and widespread speckled structures in the nuclei of infected cells, but was not detectable in purified virions. In the DEV-infected duck tissues, the UL31 antigen was primarily located in the cells of immunological organs and digestive organs. These properties of the UL31 protein provide a prerequisite for further functional analysis of this gene.

## Results and discussion

### Predicted features of the UL31 ORF

**Figure 1:**
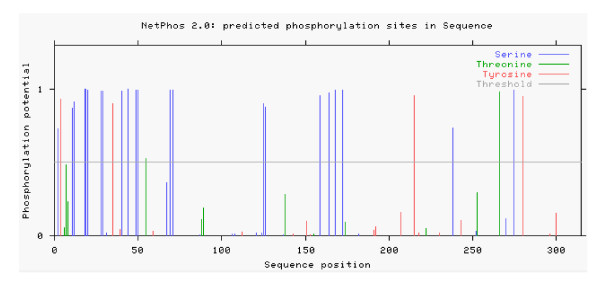
**Prediction of Phosphorylation sites of deduced amid acids of the DEV UL31 protein**. Potential sites of phosphorylation were predicted using the NetPhos algorithm available via the Expasy proteomics tools database .

**Figure 2:**
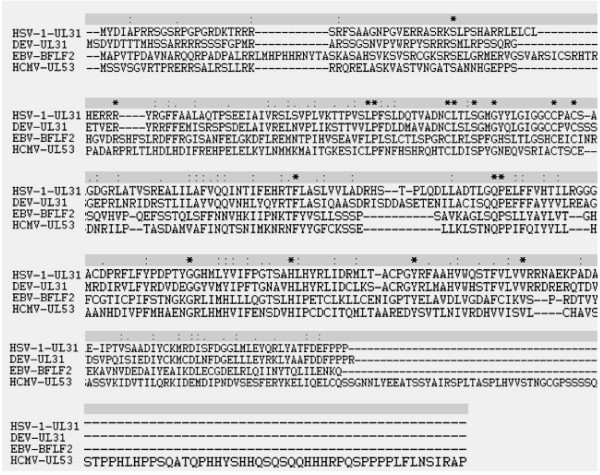
**Amino acid sequence comparison between the putative proteins encoded by DEV UL31 and it homologs in hunman herpesviruses: HSV-1 UL31, HCMV UL53, and EBV BFLF2**. Sequences were aligned with the Clustalx1.8 software. Absence of amino acid is shown by dash '-' in the sequences while '*', ':', and '.' Indicate identical amino acid residues, conserved residues and semi-conserved residues in all sequence used in the alignment respectively.

Computer analysis showed that the DEV UL31 potentially encodes a protein of 35.75 kDa, consisting of 310 amino acids and with an isoelectric point of 7.56. UL31 is predicted to be a potential nuclear localization. The sequence contains 28 possible sites for phosphorylation, 22 on serine, 2 on threonine, and 4 on tyrosine residues (Fig. 1) [21]. Furthermore, six casein kinase II, three cAMP-dependent protein kinase, four protein kinase C phosphorylation sites and one potential N-linked myristoylation site are present along the amino acid sequence. As mentioned in the introduction, UL31 has been studied extensively in human and nonhuman herpesviruses [9,19,22-24]. Fig 2, showing the UL31 family members of herpesviruses, illustrates that DEV UL31 shares identities of 37% with EBV BFLF2, 21% with HSV-1 UL31, and 19% with HCMV UL53, suggesting a potential related function.

### Expression and purification of recombinant UL31

**Figure 3:**
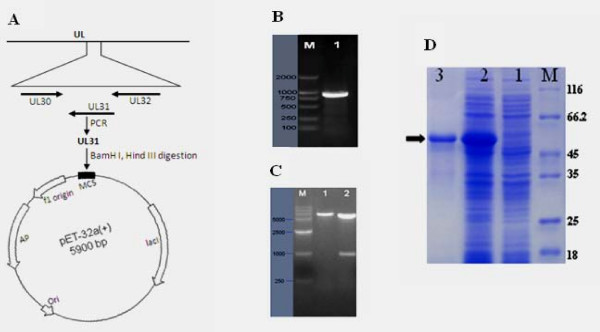
**A Schematic representation UL regions of the DEV genome containing the UL31 gene and strategy for construction of the expression plasmid pET 32-UL31**. **B **PCR product of the fragment of DEV UL31 detected by 1% agarose gel electrophoresis. Lane M, DNA marker; Lane 1, PCR product of the DEV UL31. **C **DEV UL31 gene encoding DNA sequence was cloned into pET 32a(+) prolaryotic expression vector as described in materials and methods. The construct was digested with two restriction enzymes. M, DNA marker; Lane 1, *Bam*HI generating one restriction fragment; Lane 2, *Bam*HI and *Hind*III generating two restriction fragments. **D **Induction of the His-tagged UL31 fusion protien in *E. coli*. Plasmid pET-UL31 was transformed into bacteria. Bacteria were grown in the absence (lane 1) or the presence (lane 2) of IPTG. The fusion protein was purified as described in Methods (lane 3). Molecular mass markers (in kDa) are shown to the right (lane M). The arrowhead indicates the induced UL31 fusion protein.

In the present study, DNA sequence encoding the UL31 gene was amplified from the genome of DEV (Fig 3B), and cloned into the fusion expression vector pET-32a (+) to generate the recombinant plasmid pET32-UL31, which was confirmed by restriction enzyme analysis (Fig 3C) and by DNA sequencing. To express the UL31 gene, the plasmid pET-UL31 was transformed into competent *E. coli *BL21(DE3) cells. A high level of expression of the resulting 55 kDa recombinant protein was obtained after induction for 3 h with 0.8 mM IPTG (Fig 3D, lane 2). Based on the His tag present at its N-terminal end, the recombinant UL31 was purified by Ni-NTA affinity chromatography (Fig 3D, lane 3).

### Preparation and specificity of anti-UL31 protein antiserum

**Figure 4:**
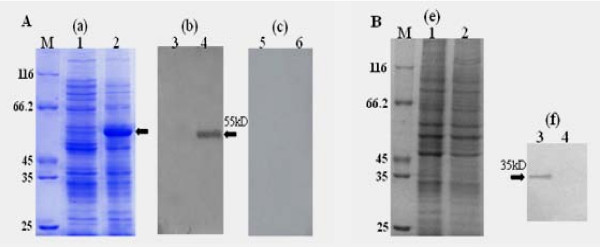
**A Expression of pET-UL31 in *E. coil *and its specificity by Western blot**. (a) *E. Coil *cells harbouring pET 32-UL31 were grown in the absence (1) or presnece (2) of IPTG. (b, c) Specificity of rabbit polyclonal antiserum against the His-UL31 fusion protein. *E. Coil *cells harbouring pET 32-UL31 were uninduced (3, 5) or induced (4, 6) with IPTG. Proteins were separated by SDS-PAGE and transferred to PVDF membranes. The membranes were incubated with the UL31 antiserum (3, 4) and preimmune rabbit serum (5, 6). Arrowheads indicate the UL31 fusion protein. **B Identifiction of the UL31 protein by Western blot**. DEF cells were mock-infected (1, 3) or infected with DEV (2, 4) and harvested at 36 h postinfection (p.i.). Proteins were separated by SDS-PAGE and stained with Coomasie brilliant blue (e). UL31 antiserum was used to identify the UL31 protein (f). Molecular mass markers (in kDa) are shown to the left (lane M). Arrowhead indicate the UL31 protein.

The anti-UL31 protein antiserum was preparation as described in Methods. Western blotting experiments were performed to examine the reactivity and specificity of the UL31 antiserum. Fig. 4A shows that the UL31 antiserum reacted with a band in the IPTG induced cell lysates with an apparent molecular mass of 55 kDa (lane 4). However, The UL31 antiserum did not react with any proteins present in uninduced cell lysates (lane 3), nor did the preimmune serum react with any proteins present in either uninduced or induced cell lysates (lanes 5, 6). Therefore, we used this polyclonal antiserum for further experiments to characterize the UL31 product of DEV.

To identify the UL31 product, SDS lysates from DEV noninfected and infected DEF cells were collected and immunoblotted with the anti-UL31 polyclonal antibody. As shown in Fig. 4B, UL31 anti-serum recognized a specific band of approximately 35 kDa in infected cell lines (lane 3). However, no signal was present in uninfected cell lines (lane 4). Nucleotide sequence analysis of coding sequences of UL31 predicts a 35.7 kDa basic protein, and thus the molecular mass of the protein reacted with the UL31 antiserum was consistent with that predicted. These results indicate that the 35 kDa protein is the product of the DEV UL31 gene.

### UL31 RNA expression in infected cells

**Figure 5:**
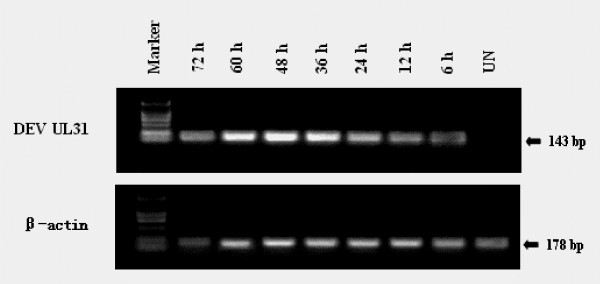
**Character of the UL31 RNA expression in infected DEF**. Total RNA was extracted from the cells for determination of UL31 mRNA expression by RT-PCR assays as described in the Methods. The upper panel shows RNA from uninfected DEF (UN) and infected DEF at different times p.i. (6, 12, 24, 36, 48, 60 and 72 h), amplified by RT-PCR. Marker: molecular mass marker DL2000 (TaKaRa). The lower panel shows β-actin, which was run as an RNA-competence control.

DEV UL31 RNA expression was analyzed by RT-PCR on total RNA. As shown in Fig. 5, the UL31 mRNA was detectable from 6 h post-infection (p.i.), was markedly increased at 48 h p.i., indicating that the UL31 gene is expressed throughout the viral replication cycle and is a not true late kinetics of expression, in agreement with data reported for its HSV-1 and ILTV homologues, UL31 [22,25]. The similar expression kinetics may be correlated with the function of the UL31 gene in different herpersviruses. PCR samples amplified without reverse transcription were negative.

### Subcellular location of the UL31 product in DEV-infected cells

**Figure 6:**
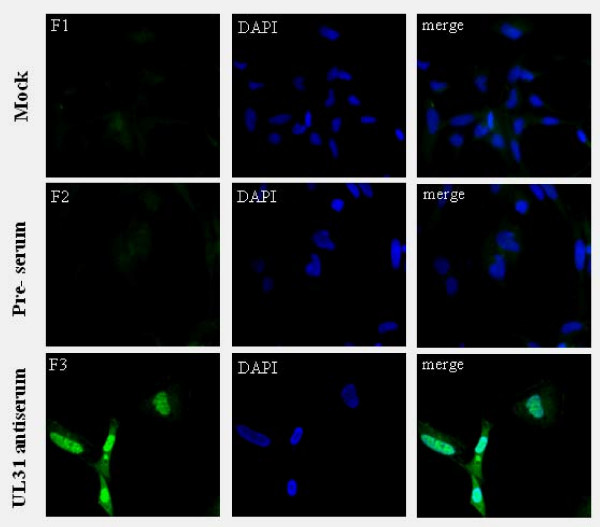
**Intracellular location of DEV UL31 protein analyzed by indirect immunofluorescence**. Mock and DEV-infected cells were fixed with 4% formaldehyde at 36 h (p.i.) and processed for indirect immunofluorescence. Mock-infected cells with UL31 antiserum (F1). DEV-infected cells with preimmune sera (F2) or UL31 antiserum (F3). The cell nuclei were visualized by DAPI. (Images were acquired by using 40× objective)

The intracellular distribution of UL31 protein was examined by indirect immunofluorescence staining. At 36 h (p.i.) postinfection, mock-infected and DEV-infected DEF cells were fixed and permeabilized as described in Methods. Then, the cells were treated with bovine serum albumin to block nonspecific binding and reacted with the UL31 antiserum. As shown in picture 6 (F3), the UL31 gene product of DEV is widespread speckled structures in the nuclei of infected cells. The homologous PRV and HSV-2 proteins exhibit similar nuclear locations, correlating with important functions during egress of viral nucleocapsids from the nucleus [14,26,27]. In contrast, no specific staining was observed in mock-infected cells that were reacted with the UL31 antiserum (Fig. 6F1) or in DEV-infected cells reacted with preimmune serum (Fig. 6F2).

### The UL31 protein was not detected in extracellular virons

**Figure 7:**
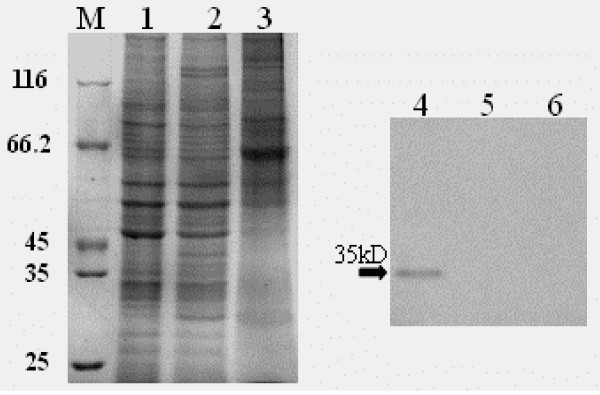
**Association of the UL31 protein with purified virions**. DEF infected (1, 4) and uninfected(2,5) cells harvested at 36 h (p.i.), purified viruses (3, 6) were separated by SDS-PAGE, and the gel was stained with Coomasie brilliant blue. Molecular mass markers (in kDa) are shown to the left (lane M). Arrowhead indicate the UL31 protein.

The above results suggest that the UL31 protein may be a component of DEV virions. To test this possibility, we next analyzed by Western blotting whether UL31 was present in extracellular virions. To this purpose, viruses from infectious supernatants obtained from the DEV-infected DEF were purified and protein extracts were analyzed by Western blotting. Fig 7 shows that no reactivity for UL31 protein was detectable in extracelluar virions with anit-UL31 bodies, whereas a strong positive signal was visible in DEV-infected cells, which is in agreement with the absence of the corresponding gene products from mature PRV or HSV-1 particles [26,28]. Although we cannot exclude the possibility that an amount of it too small to be detected is packaged in virions, these results indicated that the UL31 protein is not a component of DEV virions.

### Distribution of DEV UL31 antigen in DEV-infected ducks

**Figure 8:**
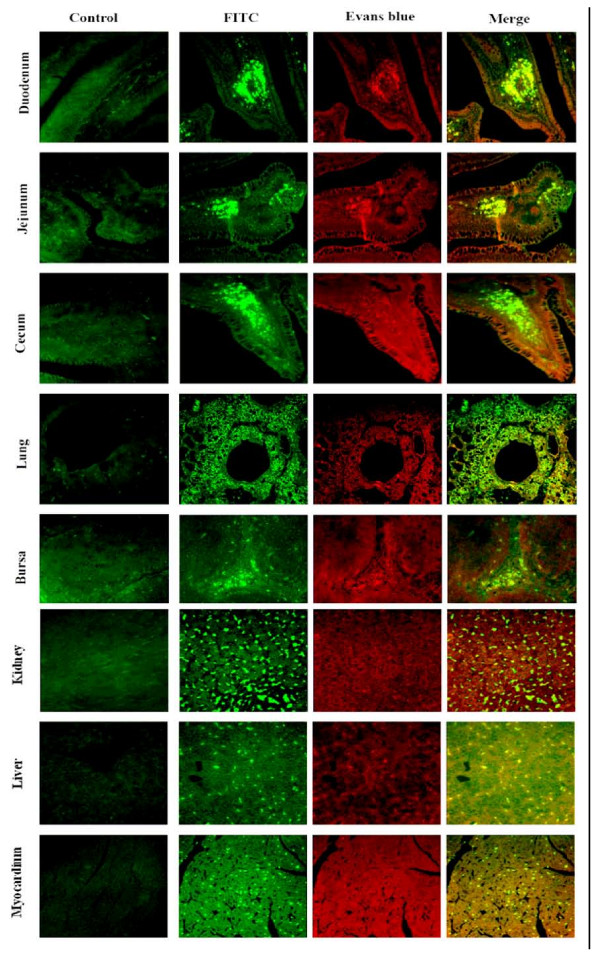
**Tissue distribution of the UL31 antigen in various organs of DEV-infected ducks**. Mock- and infected ducks were sacrificed at 4 d post-infection. Each organ was from the ducks and fixed in 4% formaldehyde solution. After fixation, the tissue samples were dehydrated and embedded in paraffin. Then the tissue sections were made at 4 μm and stained with an indirect immunofluorescent technique. Labels on the left side of the images indicate organs. Negative control is shown in the left column, and the staining methods are indicated above the top horizontal row. (Images were acquired by using 40× objective).

The distribution of DEV UL31 antigen in tissues of artificially DEV-infected ducks was studied using the immunofluorescence assay (Fig. 8). In the DEV-infected duck tissues, the UL31 antigen was primarily located in the cells of immunological organs and digestive organs such as liver, thymus, myocardiu, bursa, kindey, duodenum, jejunum, ileum, cecum, and lung. However, in the other tissues (such as Harders glands, muscle, pancreas, and cerebrum), the UL31 antigen was less positive signals (date not shown). In contrast, no positive signals were located in the tissues of mock-infected ducks. So, we conclude that the immunological and digestive organs are target organs in DEV infections of duck.

## Conclusion

In this work, the DEV UL31 gene has been successfully expressed in a prokaryotic expression system, and we present the basic properties of the DEV UL31 product. The results indicate that DEV UL31 shares many similarities with its HSV or PRV homolog UL31 and suggest that functional cross-complementation is possible between members of the *Alpha*herpesvirus subfamily. Furthermore, in vivo experiments with ducks infected with UL31-defective isolates of DEV will also be of importance in order to assess the possible role of the UL31 protein in viral pathogenesis.

## Methods

### Cells and viruses

Duck embryo fibroblasts (DEF) were grown in MEM medium (Gibco-BRL) supplemented with 10% fetal bovine serum (FBS) (Gibco-BRL), 100 units/ml penicillin and 100 μg/ml streptomycin and were used throughout this study. DEV CHv strain was a high-virulence field strain isolated from china, obtained from Key Laboratory of Animal Disease and Human Health of Sichuan Province.

### Construction of bacterial expression vector

A full-length UL31 gene was amplified by PCR from the genome of DEV CHv-strain, using synthetic oligonucleotide UL31f (5'-AAAGAATTCATGAGCCAGACCCAACCCCCG) as the forward primer and synthetic oligonucleotide UL31r (5'-TTAGTCGACTACGGCGGAGGAAACTCGTC) as the reverse primer. BamH I and Hind III sites were incorporated into the forward and reverse primers, respectively. The amplicon was cloned into a T/A cloning vector (pMD18-T Simple; TaKaRa). The UL31 sequence was subsequently released by BamH I/Hind III digestion and cloned into the Hind III and BamHI sites of pET 32a(+) (Novagen) in frame with the gene encoding His. The recombinant plasmid (pET-UL31) was confirmed by restriction enzyme digestion and DNA sequencing (TaKaRa)

### Expression and purification of UL31-His fusion proteins

The confirmed construct described above was used to chemically transform *Escherichia coli *BL21(DE3) for expression the UL31 protein. For production of UL31-His fusion protein, 100 μl of fresh stationary-phase culture was inoculated into 10 ml of Luria broth (LB) supplemented with 50 μg/ml ampicillin (Sigma). To optimize expression, the bacterial culture was grown at 37°C until the optical density at 595 nm was 0.5, at which time protein expression was induced by the addition of 0.8 mM isopropyl-β-D-thiogalactopyranoside (IPTG). The culture was shaken at 210 rpm at 37°C for 3 h in a 100 ml Erlenmeyer flask. After induction, cells were lysed in 2× sample buffer (0.1 M Tris-HCl, pH 6.8, 4% SDS, 0.2% bromophenol blue, 20% glycerol, and 0.1 M DTT) and analyzed by SDS-PAGE [29]. The recombinant His-tagged proteins were purified by nickel affinity chromatography according to the manufacturer's protocol (Bio-Rad), and analyzed by SDS-PAGE.

### Generation of polyclonal antisera in rabbits

For the preparation of polyclonal antibodies, male rabbits were immunized first with 0.5 mg of *E. coli*-expressed 6× His-tagged UL31 proteins emulsified in Freund's complete adjuvant. Inoculations were subcutaneous injections on the shaven back. Freund's incomplete adjuvant and 1 mg of purified fusion protein were used for subsequent boots. Three booster injections were given each at 1-week intervals after primary injection. Eighteen days after the last boot, blood was collected from an ear vessel. Then, sera were collected and stored at -80°C.

### Western blotting

To identify and characterize the DEV UL31 product, DEF, mock infected or infected with DEV, were harvested by centrifugation, washed once with PBS, and resuspended in PBS-1%Triton-2 M urea and briefly sonicated. Then, samples were denatured and resolved on a 12% SDS-PAGE gel and transferred onto polyvinylidene difluoride (PVDF) membrane by standard procedures [30]. For immunodetection, the membranes were blocked in 5% nonfat dry milk in PBS-T (0.2% Tween 20 in PBS, PH 7.4) for 1 h. The membranes were then washed three times with PBS-T and incubated with diluted rabbit anti-UL31 (1:200) sera for 1 h at 37°C. After three washes with PBS-T, the membranes were incubated with horseradish peroxidase-linked goat anti-rabbit immunoglobulin G (IgG) (Amersham) and specific bands were detected using an enhanced chemiluminescence (ECL system) according to the manufacturer's instructions (Amersham).

### Determination of mRNA expression of UL31 in infected cells

The levels of the mRNA transcripts of UL31 were determined by reverse transcriptase polymerase chain reaction (RT-PCR) on total RNA, extracted from uninfected or DEV-infected cells at different times p.i. (6, 12, 24, 36, 48, 60 and 72 h), using the Total RNA Isolation System (TaKaRa). The concentration of RNA was determined by measuring A260, and the purity was checked by the A260/A280 ratio (greater than 1.8). Purified RNA was treated with DNAase I (TaKaRa) and 2 μg RNA was used as template for RT-PCR. The PCR primers for UL31 cDNA and β-actin cDNA are: UL31 f (5'-GTTGCTGCCCAG TATGTT-3') and UL31 r (5'-GTCGGATGCTGCTTGTAT-3'); β-actin f (5'-CCGGGCATCGCTGA CA-3') and β-actin r (5'-GGATTCATCATACTCCTGC TTGCT-3'). cDNA equivalent of 5 ng original RNA was used in PCR. β-actin mRNA expression was determined using the same amount of cDNA as an RNA-competence control.

### Indirect immunofluorescence assays of infected cells

The DEV UL31 production location in intracellular was analyzed by Indirect immunofluorescence. DEF cells were seeded on sterile coverslips and were mock or infected with DEV. At 36 h postinfection, cells were fixed in 4% formaldehyde in phosphate-buffered saline (PBS) for 15 min at 25°C and with 0.2%(v/v) TrionX-100 in PBS for an additional 10 min at 25°C to allow permeabilization. Following several washes in PBS, cells were blocked in 5% bovine serum albumin (BSA) in PBS for 1 h at 37°C. After, The cells were reacted with rabbit anti-UL31 serum diluted 1: 200 in PBS containing 0.1% BSA for overnight at 4°C, washed three times in PBS and then reacted with 1: 100 dilution of FITC-conjugated goat anti-rabbit immunoglobulin in PBS containing 0.1% BSA for 1 h at 37°C. The cell nuclei were visualized by DAPI counter-staining (5 μg/ml, Beyotime). Fluorescent images were viewed and recorded with the Bio-Rad MRC 1024 imaging system.

### Virion purification

Biochemical characterization of extracellular virions was performed by precipitating viruses from infectious supernatants with a polyethylene glycol (PEG)-containing solution (0.5% [wt/vol] PEG 6000 in 5 M NaCl) as described previously [17,31,32]. Monolayer of DEF cells were infected with DEV and harvested from the extracellular media at 72 h postinfection by centrifugation at 10,000 × *g *for 20 min. To purify intracellular virions, lytically induced cells were extensively washed and sequentially frozen in a dry ice bath and thawed at 37°C three times. Cells were spun down at 5,000 × g for 10 min, and supernatants were filtered with a 0.45-μm-pore-size filter. Viruses present in these supernatants were further PEG precipitated as described for extracellular virions. Purified virions were analyzed by Western blotting.

### Immunofluorescence image analysis of UL31 antigen distribution

To monitor the UL31 antigen distribution in DEV infected ducks, thirty-day-old ducks (DEV free) were used. The ducks were divided into 2 groups (A and B): Group B (11 ducks) was mock-infected with PBS by intramuscular injection; Group A (22 ducks) was infected with DEV (half of LD_50_) by intramuscular injection. After 4 d post-infection, different tissues were obtained and immediately treated with 4% formaldehyde for 24 h, and then embedded in paraffin.

Four-μm thick histological sections were cut from each tissue, mounted, and baked. They were then deparaffinized and rehydrated in PBS. For antigen retrieval, the sections were treated with 0.01 mol/L citrate buffer solution (pH 6.0) for 10 min in a microwave oven. Nonspecific binding was prevented by treating the sections with 5% bovine serum albumin (BSA) at 37°C for 30 min. The sections were then treated with 1:100 diluted anti-UL31 sera for 1 h at 37°C and washed with PBS. Then, they were treated with FITC – conjugated goat anti-rabbit IgG (1:100). Slides were washed in three changes of PBS, counterstained lightly with Evans blue (EB) (0.01% for 3 min), dehydrated, and coverslipped. Images were examined under the Bio-Rad MRC 1024 imaging system.

## Competing interests

The authors declare that they have no competing interests.

## Authors' contributions

WX carried out most of the experiments and wrote the manuscript. ACC and MSW have critically revised the manuscript and the experimental design. HC, DKZ, QHL, RYJ and XYC helped in experiments. All authors read and approved the final manuscript.
